# Efficacy of Galium-68 DOTATATE PET/CT in the Detection of Metastasis Rate of Well-Differentiated Gastroenteropancreatic Neuroendocrine Tumors

**DOI:** 10.22038/AOJNMB.2019.13348

**Published:** 2019

**Authors:** Halil Komek, Tansel Ansal Balci, Canan Can

**Affiliations:** 1 Department of Nuclear Medicine, Gazi Yasargil Training and Research Hospital, Diyarbakir, Turkey; 2 Department of Nuclear Medicine, Faculty of Medicine, Firat University, Elazig, Turkey

**Keywords:** Gastroenteropancreatic (GEP), Neuroendocrine tumor (NET)PET/CT, ^ 68^Ga-DOTATATE

## Abstract

**Objective(s):**

The aim of this study was to determine metastasis rate in patients with well-differentiated grade1 (G1) and grade 2 (G2) gastroenteropancreatic neuroendocrine tumors (GEP NETs) using the gallium-68 DOTATATE positron emission tomography/computed tomography (^68^Ga-DOTATATE PET/CT). This study was also targeted toward investigating the relationship of maximum standardized uptake value (SUV_max_) with mitotic count, histological grade, and Ki-67 index.

**Methods:**

This retrospective study included 38 patients (i.e., 17 males and 21 females) with G1 or G2 GEP NETs who underwent ^68^Ga**-**DOTATATE PET/CT in Diyarbakir Gazi Yasargil training and research hospital between November 2014 and March 2018. The patients had at least one positive lesion that was approved by two nuclear medicine specialists with a minimum of 10 years of experience.

**Results:**

The median age of the patients was 50 years (age range: 27-80 years), and their mean age was 52±15 years. Out of 38 patients, 1 (2.6%), 2 (5.2%), 2 (5.2%), 3 (7.8%), 10 (28.5%), and 19 (50%) patients had primary hepatic, primary mesenteric, colon, duodenal, gastric, and pancreatic NETs, respectively. In addition, the liver metastasis, local lymph node invasions, distant lymph node metastasis, bone metastasis, peritoneal involvement, and lung metastasis were observed in 42%, 21%, 15.7%, 13%, 7.8%, and 5% of the cases, respectively. The SUV_max_ median values of G1 and G2 tumors were 9.45 (range: 4.2-43.6) and 34.9 (range: 4.1-103), respectively (P=0.003). The Ki-67 index showed a negative correlation with the SUV_max_ value of the liver metastases and the highest SUV_max_ value (P=0.001 and P=0.002, respectively). There was also a negative correlation between mitosis count and the highest SUV_max_ value (P=0.011).

**Conclusion:**

Based on the findings, although [^68^Ga]DOTATATE PET/CT is successfully used to diagnose primary GEP NETs and their metastases, the SUV_max_ value obtained from DOTATATE PET/CT showed a negative correlation with Ki-67 and mitotic count.

## Introduction

Neuroendocrine tumors (NETs) are described as a heterogeneous malignant group caused by neuroendocrine cells that can be found in many parts of the human body. Approximately 75% of the NETs originate from the gastrointestinal system and pancreas, while 25% of these tumors arise from the bronchopulmonary system or the other body regions (1).

The NETs are classified into three groups (G1, G2, and G3) according to the Ki-67 index in the 2017 World Health Organization (WHO) classification. Grade 1 (G1), G2, and G3 tumors with the Ki-67 indices of ≤ 3%, 3-20%, and 20-50% are the well-differentiated ones, and G3 tumors with a Ki-67 index of > 50% are classified as poorly differentiated neuroendocrine carcinomas (2). 

The accurate staging of NETs is of great importance as 5-year survival is directly associated with metastasis. The 5-year survival rate of NET depends on tumor localization. In this regard, patients without metastasis have a 5-year survival rate of 96.1%. This rate has been reported as 77%, 73.3%, and 50.1% in patients with lymph node, hepatic, and extrahepatic distant metastases, respectively (3, 4). 

The NETs typically express somatostatin receptor (SSTR) on the cell membranes. There are five known SSTR subtypes, the most popular subtypes of which are 2_a _and 5 (5, 6). Somatostatin analog-conjugated single-photon emission computed tomography/computed tomography (SPECT/CT) and positron emission tomography (PET)/CT radiopharmaceuticals are successfully used for diagnostic and therapeutic purposes. 

The first somatostatin analog radiopharmaceutical used for the localization of NETs is iodine-123 conjugated octreotide (7). Besides, gallium-68 (^68^Ga) DOTA-conjugated somatostatin analogs (e.g., DOTATATE, DOTATOC, and DOTANOC) are PET/CT agents preferred for NET imaging. The ^68^Ga-DOTATATE PET/CT has a higher sensitivity, compared to SSTR scintigraphy (8). Moreover, ^68^Ga-DOTATATE has a high affinity for SSTR_2_ and is a superior imaging technique, compared to other functional and anatomical imaging methods (9). The ^68^Ga-DOTATATE PET/CT facilitates the determination of patient operability by the establishment of accurate staging. This modality also allows for the assessment of the feasibility of peptide receptor radionuclide therapy (10, 11).

With this background in mind, the present study was conducted to determine the metastasis rate in patients with well-differentiated gastroenteropancreatic (GEP) NETs using ^68^Ga-DOTATATE PET/CT. This study was also targeted toward investigating the relationship of maximum standardized uptake value (SUV_max_) with mitotic count, histological grade, and Ki-67 index.

## Methods

This retrospective study was conducted in accordance with the rules and principles of the 1964 Declaration of Helsinki and its subsequent amendments. In this regard, the study was approved by Diyarbakir Gazi Yasargil training and research hospital ethics committee (No: 120/2018). This study included 38 patients (i.e., 17 males and 21 females) with G1 or G2 GEP NETs who underwent ^68^Ga-DOTATATE PET/CT in Diyarbakir Gazi Yasargil training and research hospita between November 2014 and March 2018. The participants had at least one positive lesion. On the other hand, the patients with pulmonary carcinoid, neurogenic tumor, and any G3 neuroendocrine carcinomas were excluded from the study. 

***Galium-68 DOTATATE*** ***positron emission tomography/computed tomography imaging ***

***preparation***

The ^68^Ga-DOTATATE was synthesized in a fully automated procedure compliant with good manufacturing practice using a standardized labeling sequence with ^68^Ge/^68^Ga generator (iThemba. Labs, SA), 22.5 μg (15 nmol) of DOTATATE (Technical University of Munich, Germany), and a GRP® module (SCINTOMICS GmbH, Fürstenfeldbruck, Germany) equipped with a disposable single-use cassette kit (ABX, Radeberg, Germany). After the implementation of a high-performance liquid chromatography-based quality control, 2 MBq/kg of ^68^Ga-DOTATATE was injected to each patient. 

***Positron emission tomography/computed tomography protocol***

The CT was performed with the Biograph 6 PET/CT scanner (Siemens Medical Systems, CTI, Knoxville, TN, USA) 60 min after the injection. This procedure was performed at 120 kV and 110 mA with a transaxial field of view [FOV] of 600 mm, no gap, collimation of 10Å~1.5 mm, pitch of 1.1, rotation time of 0.5 sec, slice thickness of 5 mm, 512Å~512 matrix, 3D FOV of 15.5 cm, ordered-subsets expectation-maximization iteration of 2 and subset of 8, and 5 mm full width at half maximum. 

The PET images were obtained in a craniocaudal direction with a scan time of 3 min per bed position while the patient was in a supine position with fully extended legs. All patients were administered intravenous contrast at 1.5 ml/kg before the CT. All PET/CT images were taken after voiding. Attenuation-corrected emission data were obtained using the noncontrast- or contrast-enhanced CT data extrapolated to 511 keV data.

***Image Evaluation***

Two nuclear medicine specialists with at least 10 years of experience in the field assessed the ^68^Ga-DOTATATE CT, PET, and fusion images. The SUV_max_ values ​​were calculated by plotting the regions of interest on the primary and metastatic lesions with a diameter of 2 cm. The highest SUV_max_ values ​​were calculated for each patient.

***Statistical Analysis***

The SPSS software (version 25.0; IBM Corporation, Armonk, New York, United States) was used for the analysis of the variables. Shapiro-Wilk test was used to assess the normality of distribution. Mann-Whitney U test, together with Monte Carlo simulation technique, was used to compare G1 and G2 pathology groups in terms of age, SUV_max_, and dimension values. After pathology, gender, and lymph node localization factors were brought under control, partial correlation was used to examine the correlation of Ki-67 with mitosis and other variables. 

The comparison of G1 and G2 pathology groups in terms of gender and lymph node localization values was accomplished using the Pearson Chi-square exact test. In addition, the Fisher-Freeman-Halton tests were performed using the Monte Carlo simulation technique. The quantitative values were presented as mean, standard deviation, minimum, maximum, and median. In addition, the categorical variables were expressed as number and frequency in tables. The variables were examined at a confidence level of 95%. P-value smaller than 0.05 was considered statistically significant. 

## Results

The median age of the patients was 50 years (age range: 27-80 years), and their mean age was 52±15 years (Table1). Out of the 38 patients, 1, 1, 2, 2, 3, 10, and 19 cases had jejunal, primary hepatic, colon (both of whom were operated), mesenteric primary, duodenal (1 patient was operated), gastric (1 patient was operated), and pancreatic (2 patients had pancreatic operation previously) NETs, respectively (Table 1). With regard to the tumor grading, 20 and 18 patients had G2 and G1 NETs, respectively. 

**Figure 1:**
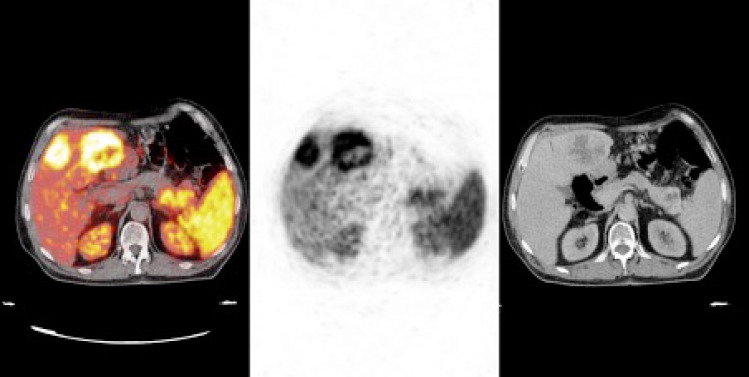
Galium-68 DOTATATE positron emission tomography/computed tomography images of a 27-year-old female patient with primary pancreatic grade 2 neuroendocrine tumor (NET) showing primary NET in the pancreas tail and metastatic foci in the liver

**Figure 2:**
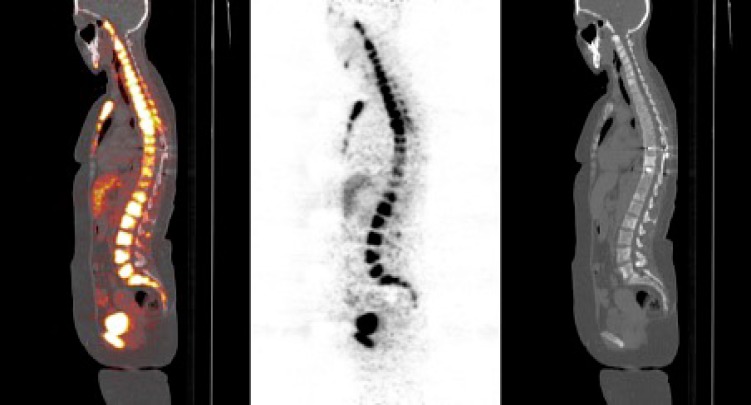
Galium-68 DOTATATE positron emission tomography/computed tomography images of a 47-year-old female patient with primary pancreatic grade 2 neuroendocrine tumor showing widespread bone metastases

**Figure 3:**
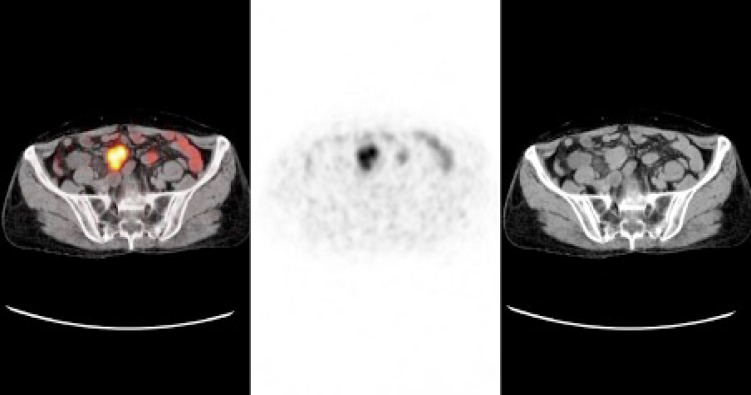
Galium-68 DOTATATE positron emission tomography/computed tomography images of a 44-year-old male patient with mesenteric grade 1 neuroendocrine tumor showing a nodular lesion with somatostatin receptor expression in the mesentery

In terms of the metastasis rate, 42%, 21%, 15.7%, 13%, 7.8%, and 5% of the cases had hepatic metastasis (Figure 1), local lymph node invasion, distant lymph node metastasis, bone metastasis (Figure 2), peritoneal involvement (Figure 3), and lung metastasis, respectively. Table 2 shows the location, size, and SUV_max_ values of primary tumors and metastases. 

Based on the results, the median values of mitotic counts in G1 and G2 tumors were 0 (range: 0-1) and 0.5 (range: 0-4), respectively (P=0.123). In addition, the highest SUV_max_ median values in G1 and G2 tumors were respectively estimated at 9.45 (range: 4.2-43.6) and 34.9 (range: 4.1-103, P=0.003; Table 3). The Ki-67 showed a negative correlation with the SUV_max_ value of hepatic metastasis and the highest SUV_max_ value (P=0.001, r=-1.00 and P=0.002, r=-0.496, respectively). Furthermore, mitosis was negatively correlated with age and the highest SUV_max_ value (P=0.011; Table 4). The rates of coexistent metastases are given in Table 5. 

## Discussion

This single-center retrospective study was aimed at determining the metastasis rate in patients with G1 or G2 GEP NETs using ^68^Ga-DOTATATE PET/CT. As another objective, the present study was targeted toward examining the relationship of SUV_max_ values in DOTATATE PET/CT with tumor grading, Ki-67, and mitosis. In a meta-analysis conducted on 22 studies with a total of 2,015 patients, ^68^Ga-DOTATOC, ^68^Ga-DOTANOC, and ^68^Ga-DOTATATE PET/CT had 93% sensitivity and 91% specificity in the detection of primary tumors (12).

The WHO has classified NETs based on the Ki-67 level that is a nuclear protein associated with cellular proliferation and mitotic count (2). The Ki-67 index is usually higher than mitotic count because the mitotic count tends to decrease during the surgical procedure and tissue fixation (13, 14). In our study, there was no significant difference between G1 and G2 tumors in terms of mitotic count. Nonetheless, Ki-67 indices were significantly different between G1 and G2 tumors. This indicates that the patients were correctly graded based on Ki-67 index.

**Table 1:** Age, pathological findings, and localization of primary tumors

		**N**	**%**
**Pathology**			
G1		18	47.4%
G2		20	52.6%
**Gender**			
Female		21	55.3%
Male		17	44.7%
**Lymph node local invasion**			
No		21	55.3%
Yes		17	44.7%
**Lymph node localization**			
None		21	55.3%
Peritumoral		9	23.7%
Subdiaphragmatic		5	13.2%
Mediastinum and neck		3	7.9%
**Localization of primary tumors**			
Pancreatic		19	50%
Gastric		10	26.3%
Duodenal		3	7.8%
Colon		2	5.2%
Mesenteric		2	5.2%
Jejunal		1	2.6%
Hepatic		1	2.6%

**Table 2:** Metastasis localization and maximum standardized uptake value of patients

	**n**	**Mean±SD**	**Median (Min/Max)**
**Age**	38	52.00±15.06	50 (27/80)
**Mitosis**	38	0.76±1.20	0 (0/4)
**Ki-67%**	38	5.50±4.23	4.5 (1/17)
**Pancreatic tumor size**	17	48.88±32.94	38 (16/133)
**Size of liver metastasis**	17	68.06±31.77	64 (14/142)
**Lymph node local invasion **	16	17.88±6.21	18.5 (6/28)
**Pancreas Suv**_max_	17	18.49±16.45	14.4 (4.2/68)
**Liver Suv**_max_	17	34.82 ± 23.53	31 (8/103)
**Lymph node Suv**_max_	16	17.02±13.35	18 (1.6/38.5)
**Primary Suv**_max_	32	16.01±13.84	11.05 (4.1/68)
**Highest Suv**_max_	38	24.48 ± of 22.05	18.45 (4.1/103)
**Gastric tumor size**	8	20.13±9.55	18.5 (7/38)
**Gastric tumor Suv**_max_	9	9.88±4.96	9.4 (4.8/21.4)
**Bone Suv**_max_	5	11.08±7.48	7.1 (4.6/22.6)
**Peritoneal metastases Suv**_max_	3	3.60±0.70	3.9 (2.8/4.1)
**Lung metastasis Suv**_max_	2	32.70±39.46	32.7 (4.8/60.6)

**Table 3.** Comparison of grade 1 and grade 2 tumors in terms of mitosis, Ki-67%, standardized uptake value, lesion size, and lymph node involvement

		**G1**	**G2**	**P-value**
		**(n=18)**	**(n=20)**	
		**n (%)**	**n (%)**	
**Gender**	Female	8 (44.4)	13 (65.0)	0.328 x
	Male	10 (55.6)	7 (35.0)	
		**Median (Min/Max)/n**	**Median (Min/Max)/n**	
**Age**		49 (30/79)/18	50 (27/80)/20	0.916 u
**Mitosis**		0 (0 / 1)/18	0.5 (0/4)/20	0.123 u
**Ki %**		2 (1/3)/18	9 (4/17)/20	**< 0.001 u**
**Pancreas size**		36 (27/133)/7	41 (16/124)/10	0.834 ^u^
**Size of liver metastasis**		69 (25/142)/4	60 (14/108)/13	0.773 ^u^
**Size of lymph node local invasion **		13 (12/21)/5	19 (6/28)/11	0.334 ^u^
**Pancreas Suv**_max_		8.5 (4.2/33)/7	17.9 (5.1/68)/10	0.054 ^u^
**Liver Suv**_max_		22 (8.5/31)/4	34 (8/103)/13	0.235 ^u^
**Lymph node Suv**_max_		5.8 (1.7/27.9)/5	21.2 (1.6/38.5)/11	0.494 ^u^
**Primary Suv**_max_		9.5 (4.2/43.6)/17	15 (4.1/68)/15	0.074 ^u^
**Highest Suv**_max_		9.45 (4.2/43.6)/18	34.9 (4.1/103)/20	**0.003 ** ^u^
**Gastric tumor size**		17.5 (7/38)/6	22 (18/26)/2	nv
**Gastric tumor Suv**_max_		8 (4.8/11.2)/6	11.4 (7.2/21.4)/3	nv
**Bone Suv**_max_		4.6 (4.6/4.6)/1	10.85 (6.5/22.6)/4	nv
**Peritoneal metastases Suv**_max_		4.1 (4.1/4.1)/1	3.35 (2.8/3.9)/2	nv
		**n (%)**	**n (%)**	
**Presence of local lymph node invasion**	Yes	12 (66.7)	9 (45.0)	0.210^ x^
	No	6 (33.3)	11 (55.0)	
**Lymph node localization**	None	12 (66.7)	9 (45.0)	0.179 ^ff^
	Peritumoral	5 (27.8)	4 (20.0)	
	Subdiaphragmatic	1 (5.6)	4 (20.0)	
	Mediastinum and neck	0 (0.0)	3 (15.0)	

**Table 4.** Correlation of Ki-67 and mitotic count with other parameters

	**Mitosis**		**Ki %**	
	**r**	**P**	**R**	**P**
**Ki %**	0.187	0.282	-	-
**Age**	-0.426	**0.011**	-0.256	0.137
**Pancreatic tumor size**	-0.009	0.976	0.434	0.121
**Size of liver metastasis**	0.205	0.482	-0.256	0.378
**Size of lymph node invasion**	-0.452	0.121	-0.465	0.109
**Pancreas Suv**_max_	-0.065	0.826	-0.232	0.425
**Liver Suv**_max_	-0.453	0.104	-1.000	**<0.001**
**Lymph node Suv**_max_	-0.445	0.128	-0.514	0.072
**Primary Suv**_max_	-0.099	0.611	-0.213	0.267
**Highest Suv**_max_	-0.425	**0.011**	-0.496	**0.002**
**Gastric tumor size**	0.264	0.667	0.070	0.911
**Gastric tumor Suv**_max_	-0.573	0.234	-0.033	0.951

Partial Correlation Test, r: Correlation Coefficient

Pathology, Gender and Lymph Node Local involvements were brought under control

Size is stated in millimeter

**Table 5:** Rates of coexistent metastases

	**Liver met** **n: 16 (%)**	**Lung met** **n: 2 (%)**	**Bone met** **n:5 (%)**	**Lymph node ** **local inv** **n: 8 (%)**	**Distant lymph node met** **n: 6 (%)**	**Peritoneal met** **n: 3 (%)**
**Liver met**	-	2 (100)	4 (80)	4 (50)	6 (100)	2 (66.6)
**Lung met**	2 (12.5)	-	1 (20)	0 (0.0)	0 (0.0)	0 (0.0)
**Bone met**	3 (18.7)	1 (50)	-	0 (0.0)	0 (0.0)	1 (33.3)
**Lymph node local invasion**	4 (25)	0 (0.0)	2 (40)	-	0 (0.0)	1 (33.3)
**Distant lymph node metastasis**	7 (43.7)	0 (0.0)	0 (0.0)	0 (0.0)	-	0 (0.0)
**Peritoneal metastasis**	2 (12.5)	0 (0.0)	1 (20)	1 (12.5)	1 (16.6)	-

Met: metastasis

Based on the evidence, there is a significant difference between well-differentiated incompatible G3 (mitotic count G2, Ki-67 G3) and poorly differentiated G3 (mitotic count G3, Ki-67 G3) tumors in terms of the survival rate (15). 

In a DOTATOC study conducted on 41 patients with GEP NET, the primary tumor sites were reported as the pancreas (29.3%), small intestine (24.4%), and stomach (2.4%); however, the primary tumor localization was unknown in 43.9% of the cases (16). In our study, 50%, 26.3%, 7.8%, 5.3%, 5.3%, 2.6%, and 2.6% of the cases had pancreatic gastric, duodenal, colon, primary mesenteric, jejunal, and primary liver NETs, respectively.

In a recent study, the examination of metastasis rate revealed that 58.4%, 51.2%, and 31.7% of the patients had hepatic, localized lymph node, and bone metastases, respectively (16). In addition, in a study conducted with DOTANOC, hepatic, lymph node, and bone metastases were respectively reported in 54.2%, 27.1%, and 6.3% of the patients, and the remaining areas had a metastasis rate of 12.5% (17). The metastasis in the mesenteric lymph nodes is the second most frequent metastasis after hepatic metastasis. 

Accurate assessment of the lymph node involvement is important in the selection of the right therapeutic method (18). In our study, liver metastasis and local lymph node invasions were observed in 42% and 21% of the cases, respectively. In addition, distant lymph node metastasis, bone metastasis, peritoneal involvement, and lung metastasis were observed in 15.7%, 13%, 7.8%, and 5% of the patients, respectively. 

Furthermore, 62%, 12.5%, 12.5%, 6%, and 6% of the liver metastases were caused by pancreatic, stomach, duodenal, colon, and primary hepatic NETs, respectively. Regarding the bone metastases, 40% of the cases resulted from pancreatic NETs, while 20% of the cases were caused by each of the colon, hepatic, and stomach NETs. In terms of the distant lymph node metastasis, 50%, 25%, and 25% of the cases originated from pancreatic, stomach, and duodenal NETs, respectively. 

With regard to the lung metastasis, 50% and 50% of the cases originated from the colon and pancreatic NETs, respectively. In a study performed by Kaewput et al., the Ki-67 showed a negative correlation with both SUV_max_ and SUV_mean_ values. In the mentioned study, a fixed value was obtained by proportioning SUV_max_ and SUV_mean_ values with the lumbar vertebras (16). Our results revealed a strong negative correlation between SUV_max_ value and Ki-67.

In the current study, the SUV_max_ values of primary tumors demonstrated no correlation with Ki-67 index and mitotic count; however, a correlation was observed between the highest SUV_max_ values with ki 67 index and mitotic count. This showed that there might be different SSTR expressions in the primary tumor and different metastatic regions of a tumor.

In a study by Kayani et al. with DOTATATE, the median SUV_max_ value of G1 tumors was higher than that of G2 tumors (19). On the contrary, in the current study, the median value of the highest SUV_max_ value in the G1 and G2 tumors were respectively 9.45 and 34.9. However, there was no significant difference between G1 and G2 tumors in terms of the SUV_max_ values of the primary tumors and metastases. In our study, the median SUV_max_ values of the liver metastases, pancreatic lesions, gastric lesions, and bone metastases were obtained as 32.5, 14.4, 18, and 7.1, respectively.

In patients with NET, CT and MRI are used for the differential diagnosis of benign and malignant lymph nodes with a short axis of less than 10 mm. The DOTATOC PET/CT is more sensitive than MRI in demonstrating metastatic lymph nodes (20). In our study, the smallest lymph node was found to be 6 mm in size.

Albanus et al., investigating PET, in addition to contrast- and noncontrast-enhanced CT, reported that that contrast-enhanced PET/CT showed higher sensitivity (92% vs. 64%) and specificity (83% vs. 59%) in comparison with noncontrast-enhanced PET/CT (21). In our study, intravenous contrast was used for all patients, which might describe the achievement of a high tumor detection ratio. The small sample size and retrospective nature of the study could be considered as the limitations of our research. 

## Conclusion

Although ^68^Ga-DOTATATE PET/CT is successfully used in the diagnosis of primary tumors and metastasis in patients with GEP NETs, in the current study, SUV_max_ values obtained from DOTATATE PET/CT showed a negative correlation with Ki-67 and mitotic count.
